# Environmental oestrogens disrupt testicular descent and damage male reproductive health: Mechanistic insight

**DOI:** 10.1111/jcmm.17837

**Published:** 2023-07-06

**Authors:** Danli Li, Hongyan Ping, Ke Li, Junjie Lin, Xuewu Jiang, Xuan Zhang

**Affiliations:** ^1^ Department of Pediatric Surgery Pingshan District Maternal & Child Healthcare Hospital of Shenzhen Shenzhen Guangdong China

**Keywords:** androgen, environmental oestrogen, infertility, INSL3, testicular dysgenesis syndrome, undescended testes

## Abstract

Environmental oestrogens (EEs) as environmental pollutants have been paid much attention due to their impact on congenital malformation of male genitourinary system. Exposure to EEs for prolonged time could hinder testicular descent and cause testicular dysgenesis syndrome. Therefore, it is urgent to understand the mechanisms by which EEs exposure disrupt testicular descent. In this review, we summarize recent advances in our understanding of the process of testicular descent, which is regulated by intricate cellular and molecular networks. Increasing numbers of the components of these networks such as CSL and INSL3 are being identified, highlighting that testicular descent is a highly orchestrated process that is essential to human reproduction and survival. The exposure to EEs would lead to the imbalanced regulation of the networks and cause testicular dysgenesis syndrome such as cryptorchidism, hypospadias, hypogonadism, poor semen quality and testicular cancer. Fortunately, the identification of the components of these networks provides us the opportunity to prevent and treat EEs induced male reproductive dysfunction. The pathways that play an important role in the regulation of testicular descent are promising targets for the treatment of testicular dysgenesis syndrome.

## 1 INTRODUCTION

Congenital malformation of male reproductive tract is one of the most common malformations at the birth.^1^ With the development of industry and environmental pollution, the incidence of congenital malformation of genitourinary system is increasing. Environmental oestrogens (EEs) as environmental pollutants have been paid much attention due to their impact on congenital malformation of male genitourinary system. EEs are external chemical pollutions caused by human activities such as industry and agriculture, and can be categorized into four groups: naturally occurring non‐steroidal plant oestrogens or phytoestrogens; steroid oestrogens—17β oestradiol and oestrone from animal and human sources; mycotoxins, zearalenone and zearalenol; synthetic compounds with phenolic groups.^2^ EEs are mainly found in pesticides, food additives, plastics and they are stable in the environment, such as sewage water used for irrigation at concentrations that could affect lucerne growth. The dietary contribution of oestrogenic industrial compounds is 0.0000025% of the daily intake of oestrogenic flavonoids in the diet.^3^

It is reported that the incidence rates of testicular germ cell tumours, low semen quality, cryptorchidism and hypospadias in male are related to prenatal environmental chemical exposure based on human and animal studies.^4^ In particular, EEs have been proved to be closely related to undescended testes (UDT) not only in epidemiological investigation, but also in many experimental studies on human and animals (Table 1).^5^, ^6^, ^7^, ^8^, ^9^, ^10^, ^11^, ^12^, ^13^, ^14^ EEs lead to a series of developmental malformations of male urogenital system due to testicular insufficiency, among which UDT is one important manifestation. Diseases related to UDT include but are not limited to cryptorchidism, sexual dysfunction, testicular atrophy, infertility and testicular tumours. Similar to oestrogen, EEs exert biological effects by binding to oestrogen receptor (ER) to regulate gene transcription in the nucleus or act in a non‐genetic manner to activate intracellular signalling pathways such as calcium and kinases in the cytoplasm to regulate cytoskeleton.^8^, ^9^, ^14^ At present, the mechanism by which EEs affect testicular descent is not completely understood.

**TABLE 1:** Environmental oestrogens (EEs) used in animal experiments.

Authors	Types of experimental animals	Ages of experimental animals	EEs	Dosage	Exposure time	Targets	Results
Schreiber et al.^1^	Pregnant rats	Sexually mature female rats	Dienestrol (DIES)	0.37; 0.75; 1.5; 3.12; and 6.25 μg/kg/day	From gestation day (GD) 6 until postnatal day (PND) 21	Male pups anogenital distance/AGD (mm) relative AGD (AGD/g b.w.) PND0/1, 4, 7, 14, and 21	AGD decrease: DIES 0.75 (PND1)/3.12 (PND1, PND21) FLU (except FLU 3.5 at PND1 and PND21) LIN (no effect) FLU+DIES FLU+LIN (except FLU 3.5 + LIN 12.5 at PND0/1) DIES + LIN (except PND4) Unable to measure due to gender discrimination barriers: FLU (12.5, 25, and 50 at PND0/1) FLU 25 + DIES 0.37 FLU 25 + DIES 3 Relative AGD lower: FLU + DIES FLU 25 + LIN 12.5 FLU 3.5 + LIN 12.5 (at PND4 and PND21) DIES + LIN (no effect)
			Linuron (LIN)	1.5; 3; 6; 12.5; 25; and 50 mg/kg/day			
			Flutamide (FLU)	3.5; 6.7; 12.5; 25; and 50 mg/kg/day		Nipple retention (NR) PND14	NR increase: DIES (no effect) LIN (no effect) FLU (100%) FLU + DIES (100%) FLU + LIN DIES + LIN (no effect)
			FLU + DIES	3.5 + 0.37; 3.5 + 3; 25 + 0.37; 25 + 3 (mg/kg/day + μg/kg/day)			
			FLU + LIN	3.5 + 12.5; 25 + 12.5 (mg/kg/day + mg/kg/day)		Cryptorchidism PND21	LIN (no effect) FLU (3.5,50) FLU 3.5 + DIES 3 (1case) FLU 3.5 + LIN 12.5 DIES+LIN (no effect)
			DIES + LIN	0.37 + 12.5; 3 + 12.5 (μg/kg/day + mg/kg/day)			
Li et al.^5^	Gubernaculum testis tissue dissected from male mice	3‐day‐old	Diethylstilbestrol (DES), insulin‐like factor 3 (INSL3)	DES (3.7 × 10^−2^ mmol·L^−1^, 3.7 × 10^−3^ mmol·L^−1^, 3.7 × 10^−4^ mmol·L^−1^, 3.7 × 10^−5^ mmol·L^−1^); INSL3 (3.3 × 10^−3^ μmol·L^−1^, 3.3 × 10^−4^ μmol·L^−1^, 3.3 × 10^−5^ μmol·L^−1^, 3.3 × 10^−6^ μmol·L^−1^)	48 h	LGR8 mRNA and protein in gubernacular cells	Significantly upregulated: DES (3.7 × 10^−2^ mmol·L^−1^)and INSL3 (3.3 × 10^−6^ μmol·L^−1^). Significantly reduced: DES (3.7 × 10^−3^ mmol·L^−1^, 3.7 × 10^−4^ mmol·L^−1^, 3.7 × 10^−5^ mmol·L^−1^)
Nef et al.^6^	Pregnant rats	E11.5, E13.5, E15.5	17α‐oestradiol; 17 β‐oestradiol; DES	17α‐oestradiol/17β‐oestradiol (6 mg); EDS (20 μg)	17α‐oestradiol/17β‐oestradiol (E13.5); DES (E11.5, E13.5, E15.5)	Male pups (E17.5, P0, P7, P20, P42)	Cryptorchidism increase (17β‐oestradiol) specific blockade of Leydig cell Insl3 gene expression (oestradiol); Insl3 expression in embryonic Leydig cells (decrease)
Mizuno et al.^7^	Pregnant rats	Cryptorchid testes in model rats during foetal stage	Flutamide	Unexplained	Unexplained	Oestrogen receptors (ERs) alpha (ERα) and beta (ERβ)	ERα (significantly decreases); ERβ (no significant differences)
Zhang et al.^8^	Kunming mice	Gubernaculum cells from 3‐ to 5‐day‐old mice	Diethylstilbestrol (DES)	DES at 0.01, 0.10, 1.00, and 10.00 μg/mL	12, 24, and 48 h	Gubernacular cells	Changed the morphology and inhibited the proliferation; intracellular [Ca^2+^] (increase); F‐Actin rearrangement and stress fibre formation
Zhang et al.^9^	Mouse	Mouse gubernacular testis cells	Diethylstilbestrol (DES)	Erk1/2 inhibitor PD98059, PKA inhibitor H89, and Src inhibitor PP2; ER inhibitor ICI 182780	—	Mouse gubernaculum testis cells	Activation of CREB downstream of PKA, Src, and ERK1/2 in these cells
Fisher et al.^10^	Pregnant rats	Gestational day (GD)13–21	Dibutyl phthalate (DBP)	0/500 mg/kg	GD13‐21	Male rats (GD 15, 17 and 19) Male pups (PND 4, 10, 15, 25 and 90 days [adults])	Testis weight (decrease); cryptorchidism and hypospadias (increase); abdominal cryptorchidism (cryptorchidism descends to the opposite side); Testicular testosterone levels (decrease, GD19, PND25); Leydig cell hyperplasia aggregation (early phase, GD17); Abnormal seminiferous cord/tubule formation (SMA decrease, GD19); Sertoli cell‐only (SCO) tubules; Evaluation of Sertoli cell maturation (AMH decrease from GD15‐19 to PND10); SCO tubules (WT‐1); Multinucleated gonocytes were detected in normal cords and in dysgenetic areas (disappeared at PND 10)
Mahood et al.^11^	Pregnant rats	Embryonic day (E13.5–21.5)	Di (n‐Butyl) Phthalate (DBP)	500 mg/kg	Embryonic day (E13.5–21.5)	Foetuses (E15.5, E17.5, E19.5, E21.5) Male pups (PND 4, 25 and 90 days [adults])	Cryptorchidism (increase); Leydig cell hyperplasia aggregation (early as E17.5, most pronounced at E21.5); testosterone levels and expression of P450 side‐chain cleavage enzyme (decrease); dysgenetic areas were present centrally in the testis (adults)
Atanassova et al.^12^	Male pups	PND 2, 4, 6, 8, 10 and 12	Diethylstilboestrol (DES)	0.1 μg DES; 0.1 μg‐DES‐GnRHa; 200 μg testosterone esters; Flutmaide; 10 μg DES; 0.1 μg DES plus either flutmaide or GnRHa	PND 2, 4, 6, 8, 10 and 12	FSH, testosterone and oestrogen (DES) levels	Increasing Sertoli cell (endogenous androgens, FSH); Decreasing Sertoli cell number (exogenous oestrogen, DES); blood inhibin‐B (evidence of Sertoli cell number)
Ping et al.^13^	Neonatal mice	GD9‐GD17	Diethylstilboestrol (DES)	2 μg/pup/day	GD9‐GD17	Gonads (gubernaculum development); proteomics analysis	Most dysregulated pathways (cardiac muscle contraction, oxidative phosphorylation, calcium signalling and cGMP‐PKG signalling pathway); enriched pathway (steroid biosynthesis)
Zhang et al.^14^	Mouse gubernaculum testis cell	‐	Phospholipase C (PLC) inhibitor U‐73122; diethylstilboestrol (DES)	DES (1.5 × 10^−8^ mol/L); U‐73122 (1 μmol/L) + DES (1.5 × 10^−8^ mol/L)	U‐73122 (10 min); gubernaculums testis cells grown to 70%–80%	Gubernaculums testis cells	U‐73122 impaired diethylstilbestrol‐evoked intracellular Ca2+ mobilisation in gubernaculum testis cells; U‐73122 inhibited diethylstilbestrol‐induced gubernaculum testis cell proliferation; U‐73122 inhibited diethylstilbestrol‐induced cAMP‐response element binding protein activation in gubernaculum testis cells

Therefore, in this review we aimed to summarize recent advances in our understanding of the role of EEs in the process of testicular descent, propose the mechanisms by which EEs affect testicular descent and highlight potential targets to block the vicious effects of EEs on genitourinary system in order to improve human health.

## 2 DEVELOPMENT OF TESTICULAR DESCENT

**FIGURE 1:**
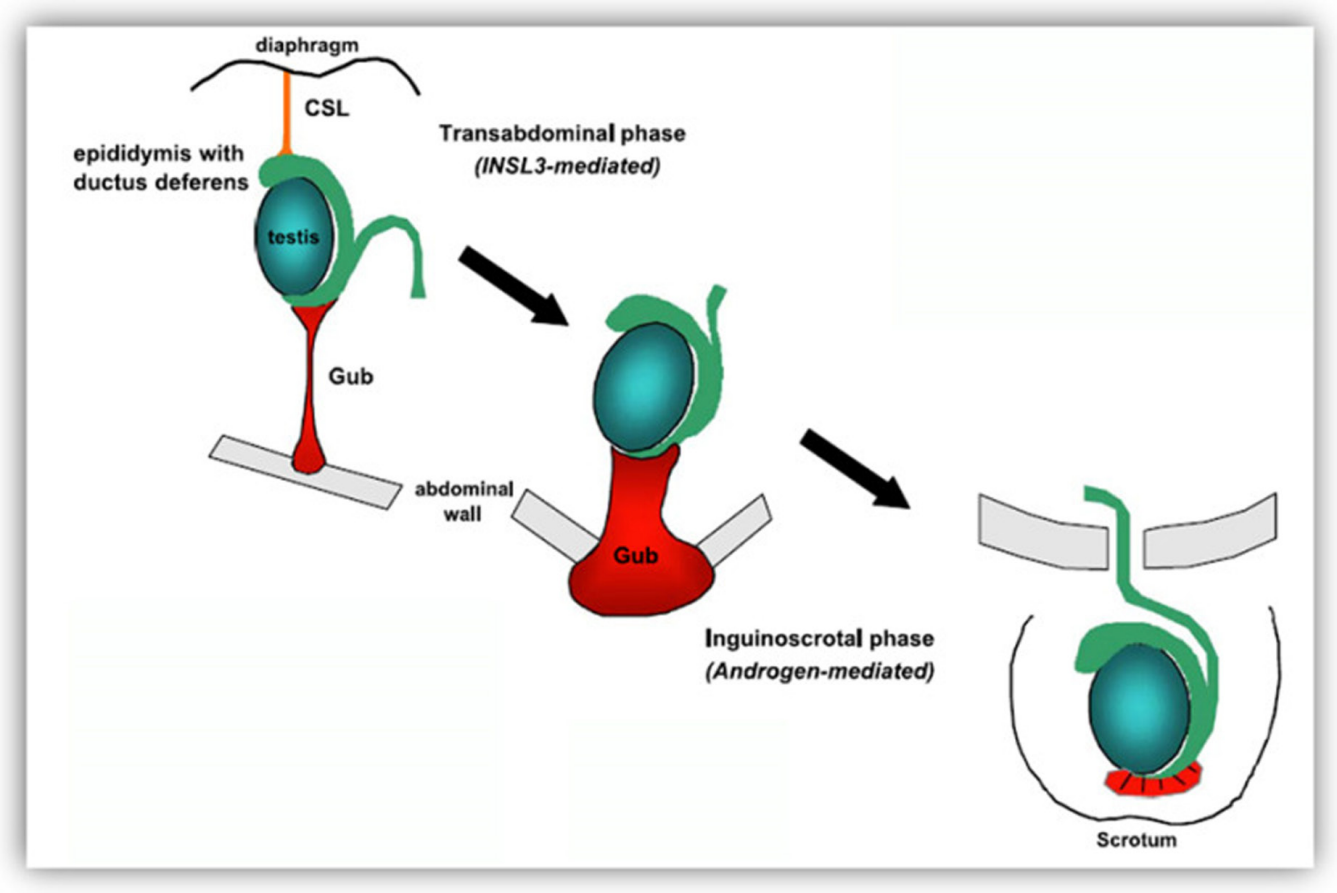
The development of testicular descent. The primitive gonad is located around the kidney, held by the cranial suspensory ligament (CSL) and the gubernaculum testis. During transabdominal phase, androgen‐mediated dissolution of the CSL and insulin‐like factor 3 (INSL3) mediated swelling of the gubernaculum would bring the testis down to the inguinal canal. Next, during inguinoscrotal phase, the testis would passe the inguinal canal into the scrotum dependent on the action of testosterone. Adapted from Ref.^15^

Testicular descent is a development process including two divided phases, transabdominal and inguinoscrotal phase (Figure 1). First, at transabdominal phase, testis originate from urogenital ridges, and begin to move to the inguinal ring at 10–15th week in human embryonic period.^15^ In the early foetus, the gubernaculum is seen as a short, thin ligament connecting the ambisexual gonad and the urogenital ridge containing Wolffian ducts (WDs) to the inguinal region.^15^ The degeneration of cranial suspensory ligament (CSL) at the top of male foetal urogenital crest, coupled with the expansion of the enlargement of the gubernaculum through mitosis and deposition of hyaluronic acid, eventually brings the testes very close to the future inner inguinal ring. This stage mainly depends on the interaction between insulin‐like factor 3 (INSL3) and its receptor.^16^ Testosterone is thought to be the sole factor responsible for the stabilisation of the WDs. Leydig cells produce testosterone at 8 weeks of gestation in humans, and testosterone is thought to be secreted directly into the WDs by diffusion.^16^ Meanwhile, androgen stimulates suspensory ligament degrade. This action will reduce the counterforce for testicular descent. The WDs then develop into separate but contiguous organs, the epididymis, vas deferens and seminal vesicles.^16^ In the inguinoscrotal phase, the gubernaculum migrates and elongates towards the scrotum, and eventually the testis descend to the scrotum. Then, at inguinoscrotal phase, testis enter into inguinal ring through groin tube, and reach scrotums. At this phase, testis experience a series of changes. For example, testis, gubernaculum and inguinal ring enlarge, spermatic veins increase and extend. This step is critically dependent on the function of testosterone.^17^

## 3 CONSEQUENCE OF UDT BY TESTICULAR UNDESCENT

Skakkebaek et al. proposed that undescended testis, hypospadias, poor semen quality and testicular cancer are the manifestations of testicular dysgenesis syndrome, named by others as ‘the developmentally estrogenized male phenotype’.^2^, ^18^ EEs also have the impact on wild animals, including cryptorchidism of Florida leopard, penile band of male otter, penile fish of alligator, sex reversal of fish and the changes in mating behaviour of birds.^19^, ^20^, ^21^ In sensitive pregnancy stage, the exposure to toxic dose of EEs would damage reproductive system, leading to testicular dysgenesis syndrome (TDS) and genitalia malformations. UDT is not only the insufficiency of testicular descent, but also directly or indirectly affects the structural and functional abnormalities of male genitourinary system, including cryptorchidism, hypospadias, testicular cancer and TDS. The declining reproductive ability and testicular tumours are two aspects that we will discuss below.

## 4 UDT DAMAGES THE FERTILITY IN MEN

In the second half of the 20th century, with the development of industrialisation, people began to realize the impact of EEs on male reproductive system. Initial studies showed that the male infertility rate in developed countries increased by 0.5%–1% every year, the proportion of cryptorchidism and hypospadias increased, and the quantity and quality of semen in industrialized countries declined.^2^ The direct observations on the human impact of oestrogen come from occupational exposure events, such as the decrease of sperm number, loss of sexual desire and impotence among workers dealing with DDT, Kepone and manufacturing DAS.^22^, ^23^ A study by American Society for Reproductive Medicine in 2014 on 149 infertile men showed that bisphenol A concentration in urine and sperm motility was negatively correlated.^24^ In addition, investigation of infertile men showed that 10% infertile men had a UDT history. Double UDT patients had six times high probability than single UDT patients.^25^ It was reported that the probability of azoospermia in single UDT patients was 13.3% and increased to 88.6% in double UDT patients. The probability of azoospermia in double UDT patients fell to 46% and 32% after treated with orchiopexy and hormone, respectively, while no significant change in single UDT patients.^26^ These results provide strong evidence that UDT damages the fertility in men.

## 5 UDT CAUSES TESTICULAR TUMORIGENESIS

Testicular cancer is the most common malignancy among men between 14 and 44 years old, and its incidence has risen over the past two decades in Western countries.^27^ Both genetic and environmental factors contribute to the occurrence of testicular cancer.^28^ EE is a risk factor for testicular tumour. A study showed that adolescents and children exposed to hormone disruptors had higher incidence of testicular cancer and seminoma.^29^ Cryptorchidism is one of the most common congenital abnormalities in boys, and is one of the few known risk factors for testicular cancer.^30^ In patients with cryptorchidism, testis development is abnormal because the testis is not located in the scrotum. The temperature of other places is higher than that in scrotum. Therefore, the risk of testicular tumour is 35–48 times higher in cryptorchidism patients than in general population. In addition, 3%–18% of cryptorchidism patients will develop testicular cancer.^31^ It was found that elevated concentrations of polychlorinated biphenyls, hexachlorobenzene and chlordanes were detected in the blood of mothers of men with testicular cancer.^32^ There is a positive correlation between household use of pesticides, especially fungicides, in early development and the risk of adult testicular germ cell tumours and non‐seminoma.^33^

## 6 THE MECHANISMS BY WHICH EEs DISRUPT TESTICULAR DESCENT

EEs do not cause instantaneous toxicity because of low concentration in the body. However, EEs can go through the blood‐placenta barrier to affect foetal development on maternal pregnancy.^34^ EEs are harmful to male genitourinary system especially during pregnant stage. EEs could hinder testicular differentiation and descent during embryonic stage. In the entire testicular descent process, many factors are involved to regulate testicular descent. EEs can interference with one or more of these factors to hinder testicular descent.^35^

## 7 IMPACT OF EEs AT TRANSABDOMINAL PHASE

As shown in Figure 1, testicular descent is mediated by INSL3 at transabdominal phase. INSL3 is secreted by Leydig cells and theca cells, and its function depends on the androgen in the body.^36^ INSL3 gene is located at 19p13.2, and INSL3 belongs to the insulin superfamily.^37^ During pregnancy, INSL3 can be detected as early as 12th week in the amniotic fluid of male foetuses, then gradually increases and reaches the peak at 15–18th week, which coincides with the transabdominal phase. INSL3 cannot be detected after 20th week.^38^ Animal experiments have shown that testis stop descending in mice after knockdown of INSL3.^39^ At the same time, the gubernaculum became small and poorly differentiated, and the testis was still located in the high abdominal cavity next to the kidney. At the 15–17th day in male mouse embryo, the gubernaculum tail enlarged as big as testicle and led testicle into the groin area.^39^

EEs inhibit testicular INSL3 expression and change the contractibility of gubernaculum. Many animal experiments have proved that pregnant rats were distracted by environment endocrine interference contamination such as phthalic acid esters (DEHP) or diethylstilbestrol (DES) and INSL3 expression decreased significantly.^5^ Prenatal exposure to oestrogens downregulated INSL3 expression and led to cryptorchidism in mice.^6^ Excessive oestrogen would antagonize the androgen in the body and therefore indirectly affect the differentiation of Leydig cells.^40^

## 8 IMPACT OF EEs ON GUBERNACULUM

Even small doses of EEs can significantly affect the gubernaculum, including morphological abnormalities, gubernaculum cell proliferation and small myofibrils in the muscle cells.^40^ EEs cause the changes in gubernaculum cell ultrastructure and the expression of actin.^7^ Gubernaculum contains muscle composition which can shrink and tract testicular descent and plays a key role in the testicular descent process.^41^ Actin plays an important role in contraction activities because it is the main components of thin filament.^42^ Actin meditates many important biology function, including cell motility, migration and signal transmission. Our previous studies showed that oestrogen reduced actin expression in gubernaculum cells, inhibited the structure and contractile activity of gubernaculum cells and impaired testicular descent. Meanwhile, gubernaculum express androgen receptors, oestrogen receptors (AR and ER) and the G protein‐coupled oestrogen receptors (GPER). DES could impede gubernaculum differentiation through ERK1/2 signalling pathway.^8^, ^9^ Therefore, EEs could impair the process of testicular descent by inhibiting the development of gubernaculum during pregnancy.

## 9 IMPACT OF EES AT INGUINOSCROTAL PHASE

In androgen insensitivity syndrome patients and in animals with anti‐androgen treatment, testicular descent failed in inguinoscrotal stage.^43^, ^44^ From endocrinology perspective, testicular Leydig cell secrete INSL3 and androgen by luteinising hormone (LH) and Sertoli cell secrete androgen‐binding protein (ABP) by follicle stimulate hormone (FSH).^45^ Testosterone combines with AR to act on genitofemoral nerve (GNF) releasing calcitonin gene‐related peptide (CGRP). CGRP then binds to the receptors on the gubernaculum, and the gubernaculum shrink and lead testicular into scrotum to complete the process of testicular descent.

## 10 IMPACT OF EES ON TESTOSTERONE METABOLISM

Testosterone (T) is the most common androgen. The expression of P450 17α hydroxylase in testicular Leydig cell of male foetal rats decreased after the female rats were treated by EEs during pregnancy.^46^ Therefore, EEs could inhibit cytopigments P450 (CYP450) to reduce the production of T. Phthalates increase the production of testicular testosterone in foetal rats (low‐dose effect), but high dose will reduce the production of testicular testosterone in foetal rats, resulting in a lower incidence of anogenital distance and increased incidence of cryptorchidism.^47^ Some studies found that EEs could suppress 5α‐NADPH activation in the metabolism of T. In addition, T converts to oestradiol (E2) under the action of aromatase. In testicular cells, too much oestrogen can directly inhibit steroid hormones synthesis, including androgen. Disruption of steroidogenesis in FLCs by environmental xenobiotics and/or metabolic endogenous factors caused undermasculinisation and malformation of the male reproductive organs.^48^

## 11 IMPACTS OF EES ON TESTICULAR LEYDIG CELLS

Leydig cells are an important source of androgen processing. Animal experiments indicated that dibutyl phthalate (DBP) disrupted rat testicular Leydig cell function and decreased the levels of T and INSL3, leading to increased incidence of cryptorchidism and hypospadias.^10^ Image analysis showed that testicular Leydig cells got together before birth after DBP treatment. This phenomenon is considered the main feature of testicular hypoplasia.^11^ Therefore, EEs have inhibitory effects on Leydig cell development.

## 12 IMPACTS OF EES ON TESTICULAR SERTOLI CELLS

Sertoli cells secrete ABP with the help of FSH. Flutamide, an AR antagonist, could reduce the quantity of Sertoli cells via interference with the androgen in neonatal rats.^12^ The quantity of Sertoli cells significantly reduced with decreased synthesis and secretion of ABP. The quantity of testicular Sertoli cells will determine how many germ stem cells develop to sperms.^49^, ^50^ In vitro studies of Sertoli cells demonstrated altered gap junctions and impaired intercellular communications by affecting the distribution of connexion 43 (Cx43) and zona occludens‐1 (ZO‐1).^48^ Maternal exposure to BPA was shown to decrease the efficiency of spermatogenesis and sperm production in male offspring.^48^ As a result, EEs affect the quantity of sperm formation.

## 13 SUMMARY

**FIGURE 2:**
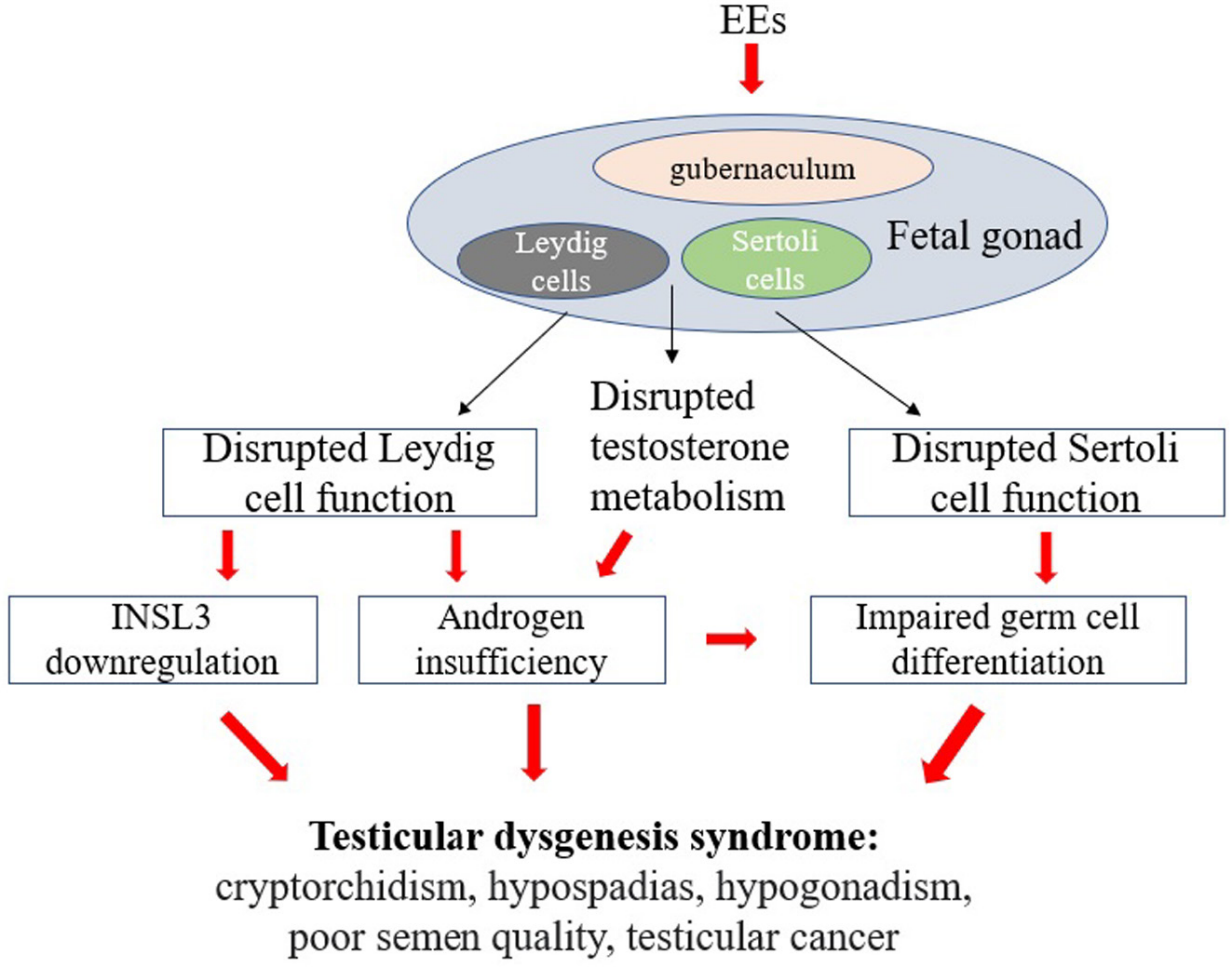
Proposed mechanisms by which EEs disrupt testicular descent and damage male reproductive health. The exposure to EEs during pregnancy causes damage to the gubernaculum, Leydig cells and Sertoli cells in the foetal gonad, and disrupt the metabolism of testosterone. Consequently, the foetus will develop testicular dysgenesis syndrome such as cryptorchidism, hypospadias, hypogonadism, poor semen quality and testicular cancer.

Exposure to EEs for prolonged time could hinder testicular descent and cause testicular dysgenesis syndrome. For male individuals, the incidence of reproductive diseases related to UDT keeps an increase trend in recent decades. Therefore, it is urgent to understand the mechanisms by which EEs exposure disrupt testicular descent. In this review, we summarize recent advances in our understanding of the process of testicular descent, which is regulated by intricate cellular and molecular networks. Increasing numbers of the components of these networks such as CSL and INSL3 are being identified, highlighting that testicular descent is a highly orchestrated process that is essential to human reproduction and survival. The exposure to EEs would lead to the imbalanced regulation of the networks and cause testicular dysgenesis syndrome such as cryptorchidism, hypospadias, hypogonadism, poor semen quality and testicular cancer (Figure 2).

Fortunately, the identification of the components of these networks provides us the opportunity to prevent and treat EEs induced male reproductive dysfunction. For example, our recent studies have identified the proteome involved in diethylstilbestrol induced dysfunction of testicular gubernaculum, among which calcium signalling pathways are crucial to the regulation of testicular gubernaculum development.^13^, ^14^ Therefore, the pathways that play an important role in the regulation of testicular descent are promising targets for the treatment of testicular dysgenesis syndrome.

## AUTHOR CONTRIBUTIONS

**Danli Li:** Data curation (equal); writing – original draft (equal). **Hongyan Ping:** Data curation (equal); writing – original draft (equal). **Ke Li:** Data curation (equal); writing – original draft (equal). **Junjie Lin:** Data curation (equal); writing – original draft (equal). **Xuewu Jiang:** Data curation (equal); writing – original draft (equal). **Xuan Zhang:** Conceptualization (equal); funding acquisition (equal); writing – review and editing (equal).

## FUNDING INFORMATION

This study was supported by the Science and Technology Innovation Commission of Shenzhen (JCYJ20190809145809375), Guangdong Basic and Applied Basic Research Foundation (2021A1515010009), Project of Educational Commission of Guangdong Province of China (2020KQNCX011) and Pingshan Health System Research Foundation of Shenzhen (202270).

## CONFLICT OF INTEREST STATEMENT

The authors declare no conflict of interest.

## Data Availability

Data sharing is not applicable to this article as no new data were created or analyzed in this study.
